# Treatment of Conjunctivitis

**Published:** 1907-07-27

**Authors:** 


### OPHTHALMOLOGY.

### TREATMENT OF CONJUNCTIVITIS.

### I. REMARKS ON TREATMENT.

THE essential feature of conjunctivitis is an abnormal secretion. Apart from trauma or chemical irritation, this is almost invariably due to the invasion of the conjunctiva, when its power of resistance is lowered, by some micro-organism.

The clinical classification of the disease, namely, "catarrhal, muco-purulent, and purulent," depends on the nature of the discharge. An accurate diagnosis can be made only by a bacteriological examination; and as a knowledge of the micro-organism is a valuable aid to treatment, such an examination should always be made. It is usually sufficient to make a cover-glass preparation, fixing the discharge by passing over the flame of a spirit lamp, then staining with methylene blue. Another film may be stained by Gram's method. In some cases it is necessary to make cultures and inoculation experiments.

The organisms usually found are:—

| Clinical Variety | Nature of Discharge | Micro-organism |
|---|---|---|
| CATARRHAL: | | |
| Acute or chronic | Muco-purulent | Koch-Week's bacillus, pneumococcus, streptococcus, staphylococcus aureus et albus |
| FOLLICULAR: | | |
| Angular, subacute and prone to relapse | Usually scanty | Morax-Axenfeld diplobacillus |
| Purulent | Pus | Most frequently, Neisser's gonococcus |
| MEMBRANOUS | Purulent | Klebs-Loeffler bacillus, streptococcus, staphylococcus, pneumococcus |

The eyes must on no account be bandaged, but left uncovered for the free escape of secretion. The discharge is very contagious, and it is the duty of the practitioner to see that others are protected. If only one eye is infected it is of the utmost importance to prevent the spread of the process to the other one. Buller's shield is one of the best devices for securing this end.

A child ought not to go to school, and should be kept at home, but not indoors unless the discharge is profuse or complications are present. Towels, sponges or face cloths, and tooth-brushes should be set aside for the sole use of the patient. If possible, personal ablutions should be performed in running water to prevent the possibility of the reintroduction into the conjunctival sac of any discharge washed out. Infection of others, and reinfection of the patient, in the treatment of the conjunctiva, are to be guarded against by the sterilisation of droppers, glass ointment rods, etc., by the use of cotton wool pellets only once, by their immediate destruction after use, and by the cleanliness of the doctor's hands.

The conjunctival sac should be well washed out with an antiseptic lotion. In mild cases a lotion of boracic acid is usually sufficient, and the application of a simple ointment or vaseline to the eyelids at bedtime prevents the discomfort caused by the gumming of the lids in the morning and permits of a free drainage. In severer cases lotio hydrarg. perchlor (1 in 6,000) is to be used, three or four times a day, and in the intervals the discharge should be frequently wiped away with pellets of cotton wool wrung out in the lotion.

Once a day, the best time being the morning, a solution of silver nitrate, 10 grains to 1 oz., should be applied to the conjunctiva, the lids being previously everted, with a piece of wool twisted on a glass rod or wool holder, it being observed that the same piece of wool must not be used for the two eyes. A stronger solution of silver nitrate is unnecessary, and need never be used. Cold sponging or iced compresses relieve the irritation consequent on the application. A solution of protargol 20 per cent., or argyrol of the same strength, may be used instead of the silver nitrate. The use of eye drops of silver nitrate, grs. ii. of zinc sulphate grs. ii. to iv., of alum grs. iv., etc., to 1 oz. distilled water, is not nearly so beneficial as this method. When there is little or no discharge drops may be used.

In the "Morax-Axenfeld diplo-bacillary conjunctivitis" sulphate of zinc is indicated. This may be used as a lotion, 1 to 10 grains to 1 oz.—the stronger solutions being used in the more obstinate cases. As a rule the discharge is scanty, and then gr. ij. ad ℥j. is strong enough. At the outer and inner canthi, at the junction of skin and conjunctiva, the lids frequently present a red and macerated surface, and from this typical condition the name "angular conjunctivitis" arises. The application to the lids of zinc oxide or boracic acid ointments furthers a cure.

The nasal condition, being a source of auto-infection, is worthy of more attention than it receives. A bacteriological examination of the nasal discharge reveals the fact that some micro-organisms are present, frequently in considerable numbers, as in a pure culture.

The Morax-Axenfeld diplo-bacillus is present in 50 per cent. of the nasal discharges; in 90 per cent. of these cases it is present in the conjunctival sac also. Diplococci are present in 30 per cent., streptococci, staphylococci, and mixed infections in 20 per cent.; these organisms are found at the same time in the discharge from the eye, in the same proportion as the diplo-bacillus is. Children especially use the same handkerchief to blow the nose and to wipe the eyes, and so an auto-infection is established. For this reason the nares should be treated at the same time as any conjunctivitis.

The application of the following ointment to the nasal mucous membrane has been found very useful, shortening the duration of the conjunctivitis considerably:—

Hydrarg. perchlor. ... ... ... gr. $\frac{1}{16}$

Acid. carbolic. (crystals) ... ... ʒj.

Ung. zinc. oxidi ad ... ... ... ℥j.

The ointment to be used twice a day.

